# Local and global features of genetic networks supporting a phenotypic switch

**DOI:** 10.1371/journal.pone.0238433

**Published:** 2020-09-03

**Authors:** Aseel Shomar, Omri Barak, Naama Brenner

**Affiliations:** 1 Department of Chemical Engineering, Technion, Haifa, Israel; 2 Network Biology Research Laboratories, Lorry Lokey Center for Life Sciences and Engineering, Technion, Haifa, Israel; 3 Rappaport Faculty of Medicine, Technion, Haifa, Israel; University of Colorado Boulder, UNITED STATES

## Abstract

Phenotypic switches are associated with alterations in the cell’s gene expression profile and are vital to many aspects of biology. Previous studies have identified local motifs of the genetic regulatory network that could underlie such switches. Recent advancements allowed the study of networks at the global, many-gene, level; however, the relationship between the local and global scales in giving rise to phenotypic switches remains elusive. In this work, we studied the epithelial-mesenchymal transition (EMT) using a gene regulatory network model. This model supports two clusters of stable steady-states identified with the epithelial and mesenchymal phenotypes, and a range of intermediate less stable hybrid states, whose importance in cancer has been recently highlighted. Using an array of network perturbations and quantifying the resulting landscape, we investigated how features of the network at different levels give rise to these landscape properties. We found that local connectivity patterns affect the landscape in a mostly incremental manner; in particular, a specific previously identified double-negative feedback motif is not required when embedded in the full network, because the landscape is maintained at a global level. Nevertheless, despite the distributed nature of the switch, it is possible to find combinations of a few local changes that disrupt it. At the level of network architecture, we identified a crucial role for peripheral genes that act as incoming signals to the network in creating clusters of states. Such incoming signals are a signature of modularity and are expected to appear also in other biological networks. Hybrid states between epithelial and mesenchymal arise in the model due to barriers in the interaction between genes, causing hysteresis at all connections. Our results suggest emergent switches can neither be pinpointed to local motifs, nor do they arise as typical properties of random network ensembles. Rather, they arise through an interplay between the nature of local interactions, and the core-periphery structure induced by the modularity of the cell.

## Introduction

Cells undergo transitions between different functional phenotypes in response to environmental cues. Although the cell is a complex high-dimensional system, much focus has been placed on the role of small, few-component circuits in such transitions. Such network motifs have been identified both in transcriptional and post-transcriptional regulation [1, 2]. When isolated, they have functionalities which may correspond to those of the entire cell state, and thus provide an intuitive molecular-scale description of cell state transitions.

However, the relationship between local and global properties of the network in giving rise to cell-state transitions remains elusive. How do multiple local motifs upscale their functionality to the entire network? How sensitive is this upscaling to network global connectivity properties, and to the features of gene-gene interactions?

A prominent, well studied switch arising both in development and in cancer progression is the epithelial-mesenchymal transition (EMT) [3, 4]. EMT is a reversible process by which epithelial cells, which normally adhere to each other, acquire mesenchymal properties, such as enhanced migratory capacity and invasiveness. Underlying this reversible process is a bistable landscape corresponding to the two phenotypes. At the local level, previous studies showed how such bistability could arise from a double negative motif in which two molecular components known to play important roles in the process, ZEB1 and miR200, mutually inhibit each other [5, 6]. However, as in other biological switches, the picture is more complex than a switch comprised of a two-component circuit. First, EMT is not a binary switch; cells can attain various states arrayed along the epithelial–mesenchymal spectrum of phenotypes. These states are called hybrid epithelial/mesenchymal phenotypes and are associated with poor prognosis in cancer due to their combined invasiveness and intercellular adhesion [3, 4].

Second, other local motifs have also been proposed to contribute to controlling the transition [7]. How the logic of multiple coupled motifs controls the entire cell-state is not well understood. Finally, experimental evidence suggests the involvement of the ZEB1-miR200 motif in other cell-state transitions such as the stemness-differentiation switch, proliferation-growth arrest switch and more [8]. Thus, while the participation of the identified molecular components in these processes is well established, there is no one-to-one causal relation between the local structures and motifs and the global cellular behavior.

Mathematical network modeling is a powerful approach to decipher the contributing factors to cell-state transitions, their interactions and logic. The EMT transition, in particular, has been the topic of many such modeling studies both at the local and at the global levels [9, 10]. These models generally include a combination of transcriptional and post-transcriptional regulatory elements [11]. Small motif models have been developed which describe the two-state landscape [12–15]. Some models are tristable, by construction giving rise to stable E/M states and a hybrid state. Recent advancements in genome, transcriptome and proteome measurements have opened the door to modeling on a broader, network-wide scale [16–19]. Bistable clusters of phenotypes with intermediate less stable hybrid states were captured by a Boolean dynamical model of the EMT network [17]. It was proposed that the emergence of multiple hybrid states stems from the similarity of the network model to a spin glass.

In this work, we utilize the above Boolean model as a case study of the relationship between the local and emergent system properties. We examine network properties on three levels: the single gene response function, the contribution of local connectivity, including the double negative feedback loop, and the global network structure. We find that hysteresis at the single gene level and the decomposition of the network into a core and periphery are the main factors contributing to the existence of two clusters of states and hybrid states. In contrast, details of connectivity, such as the double negative motif, are not essential. We thus conclude that the functionality of local motifs is not necessarily retained when they are embedded in the network level. Nevertheless, the network’s phenotypes are not independent of its structure, as both global randomization of the structure and a few well-chosen local changes can destroy its two-cluster landscape.

## Results

**Fig 1:**
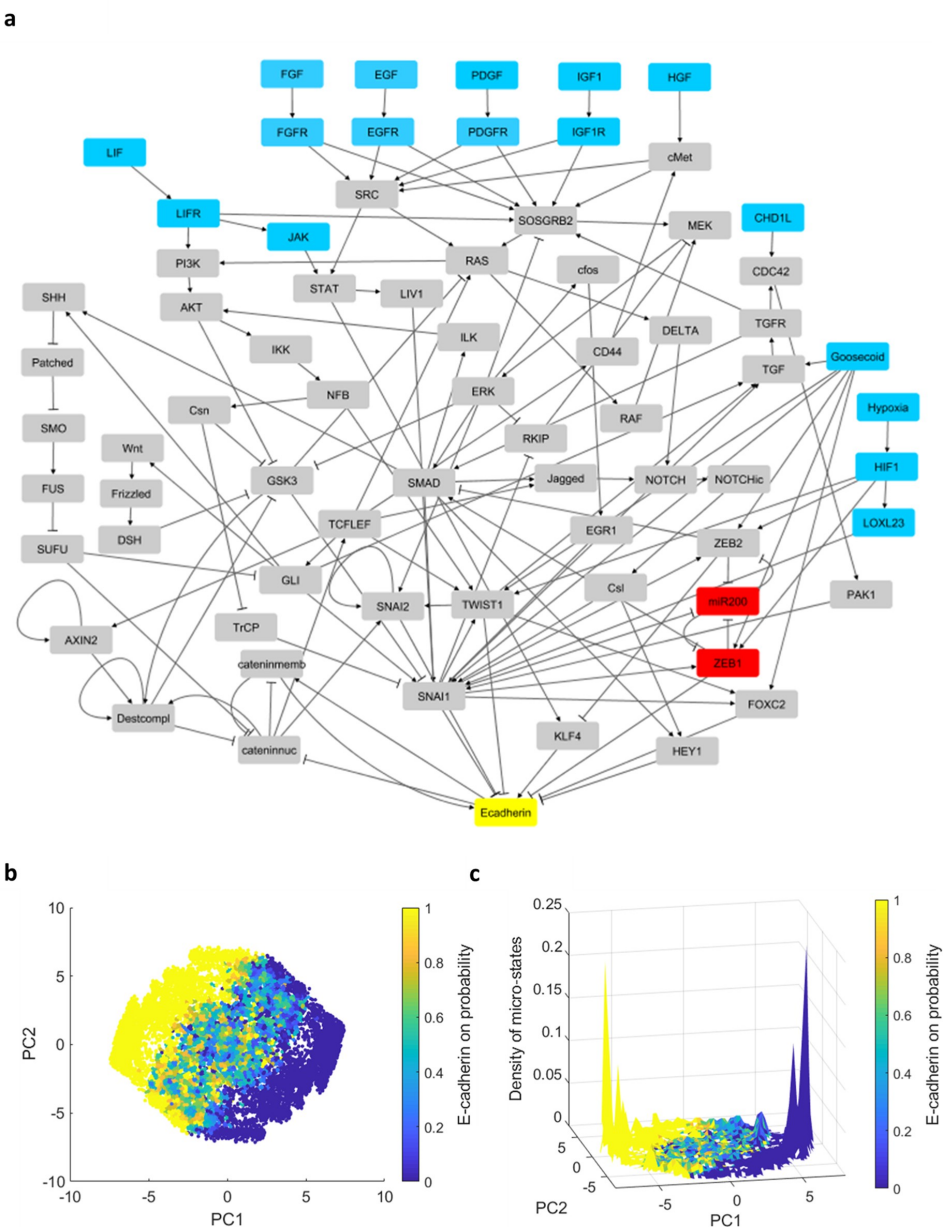
The EMT network and the topography of the epithelial and mesenchymal states. **(a)** The 72 nodes, 142 edges network model of the EMT process [17, 18]. Nodes represent molecular elements and edges represent activating (arrows) and inhibitory (caps) relationships between the nodes. E-cadherin, one of the main epithelial markers, is highlighted in yellow. The ZEB1/miR-200 double negative motif is highlighted in red. Input nodes that do not participate in the dynamics are highlighted in blue and define the network 'periphery', while the rest form its 'core' of interconnected nodes). The network illustration was produced using Cytoscape software. **(b-c)** Fixed points of the Boolean model associated with the network in (a). 38000 simulations were performed starting from random initial conditions; fixed points in 72-dimensional gene expression space were projected and binned on the first two principal components. **(b)** Distribution of fixed points in PC space, with colorbar denoting the fraction of states within each bin that express E-cadherin. PC1 explains 37% of the variance, whereas PC2 explains 11% of the variance. **(c)** Color as in (b); z-axis shows the density of micro-states. Around the two peaks, pure epithelial and mesenchymal states (1 and 0 fraction of E-cadherin expression) are found. They are connected by a rugged valley of intermediate states that corresponds to hybrid E/M states. These results reproduce those of [17].

To understand how local and global features of genetic networks support a phenotypic switch, we analyzed a model of the EMT network that gives rise to bistable clusters of E/M states intervened by less stable hybrid states. This network was constructed following previous work based on experimental data [17, 18]. It is composed of 72 molecular elements (nodes; we will also refer to these as genes, although some of them represent small miRNA or even environmental conditions) with 142 connections (edges) between them (Fig 1A). Each edge represents either an activating or an inhibitory relationship between the molecular elements represented by the nodes. Overall, the network contains 107 activating (inducing) and 35 inhibitory (repressing) connections that underlie interactions induced by growth factors, signal transduction pathways and transcriptional regulators. All these processes converge to regulate the expression of E-cadherin, which is induced in the epithelial state, keeping the cells adhered to each other, and repressed in the mesenchymal state.

A simple model for the dynamics in the network is based on Boolean functions. Such dynamics have been extensively used to model gene regulation networks [20–26]. In particular, for the EMT network, Boolean threshold dynamics were shown to capture well the bistable clusters of E/M states and less stable hybrid states [17]. In this approach the state of the network is defined by a vector of binary variables {*s*_*i*_}, determining whether each gene is expressed (*s*_*i*_ = 1) or repressed (*s*_*i*_ = −1). Regulatory relations between the genes are represented by a connectivity matrix *J*_*ij*_ where *J*_*ij*_ = 1 if gene *j* promotes gene *i*, and *J*_*ij*_ = −1 for inhibition. The states of the genes are updated asynchronously according to a majority rule: If the input to a gene is positive in total, the node becomes active (*s*_*i*_ = 1). If it is negative, the gene state is set to *s*_*i*_ = −1. Thus, the update rule can be expressed as

$$s_{i}(t+1)=sign\left(\sum_{j}J_{ij}s_{j}(t)\right)$$ (1)

In case of ties (input sum equals zero), the gene state is not updated, and retains its present state.

Simulating these dynamics over a large number (10^7^) of random initial conditions gives rise to a vast number of distinct binary steady states [17]. The distribution of states can be visualized by projecting them on a two-dimensional map using principal component analysis (PCA). These micro-states cluster into two groups connected by intermediate states, displaying a rugged and complex landscape (Fig 1B and 1C). Moreover, marking them with the fraction of steady states that express E-cadherin reveals that these two clusters correspond to high and low fractions of E-cadherin expressing states, typically categorized as epithelial and mesenchymal phenotypes, respectively. This suggests that the vast number of “micro-states”, differing in their exact pattern of gene expression, support two phenotypes. The intermediate states correspond to the hybrid E/M phenotype which combines properties from both. This is manifested in the model as an intermediate fraction of E-cadherin expressing states. Such states were recently suggested to characterize mobile cell clusters that may play an important role in metastasis [12].

These intriguing observations are consistent with the idea that a cell state is not a single gene-expression pattern but rather a cluster of such patterns that share phenotypic properties. While traditionally cell type was associated with fixed point attractors of Boolean networks [23], more recently it was suggested that they should rather be associated with a cluster of attractors by considering the effect of noise in the system [27]. The clusters found in [17] were obtained from Boolean dynamics without noise, and were interpreted as representing a complex, glass-like property of the network itself. Such a complex landscape of steady states is not a typical feature of random Boolean networks [28]; it is important to understand whether it is typical of biological networks. Therefore, we aimed to further clarify the properties of the EMT model giving rise to two clusters of states and a large number of hybrid states. Is the EMT network special in its architecture? Does the double-negative motif scale-up to induce a macroscopic form of bistability on the entire network? How does the Boolean dynamics itself affect the landscape? We used simulations of the model with specific modifications to address these questions.

### Eliminating double negative motifs does not change E/M topography

**Fig 2:**
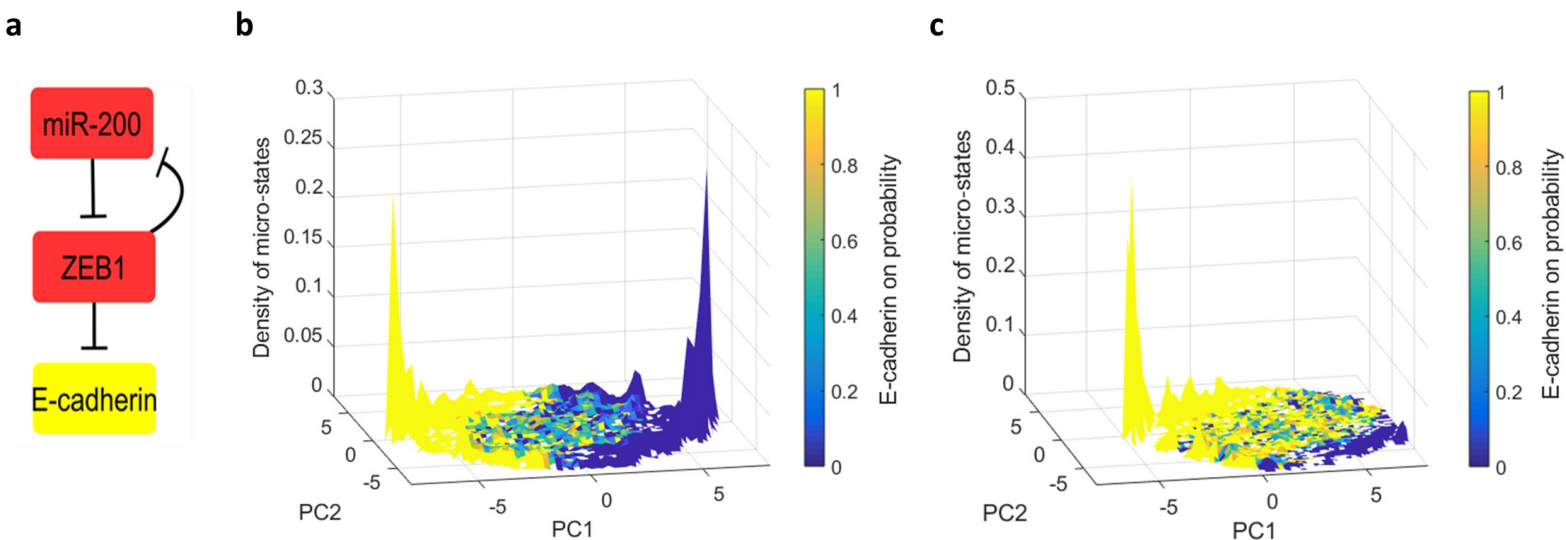
The effect of the double negative motif on the emergence of two clusters of states. **(a)** The double negative motif ZEB1/ miR200. ZEB1 inhibits E-cadherin. **(b)** The density of micro-states obtained from the EMT network after turning the inhibitory connection from ZEB1 to miR-200 into a promoting connection. Variance explained by PC1 and PC2 are 38%,11% respectively. Compare to Fig 1C. **(c)** Following ZEB1 knockdown (fixation at -1) the landscape exhibits a significant shift towards the E states. Variance explained by PC1 and PC2 are 33%,12% respectively.

First, we sought to test the effect of the known ZEB1/miR200 motif on the phenotypic switch within the network model (Fig 2A). ZEB1 inhibits E-cadherin, and its participation in the double-negative loop helps to stabilize E-cadherin at either a high or a low expression state. Simulating the Boolean dynamics on this circuit alone results in two stable fixed points [12–14]. One is identified with the epithelial state where E-cadherin and miR200 are expressed while ZEB1 is repressed, and the opposite with a mesenchymal state.

Although the isolated double negative motif by itself gives rise to bistable E/M states, this is not necessarily true when it is embedded in a large complex network. To test the necessity of the double negative motif within the network context, we turned the inhibitory connection from ZEB1 to miR-200 in the EMT network into a promoting connection. Although this destroys the isolated double-negative circuit, no tangible changes in the landscape of micro-states were observed (compare Fig 1C with Fig 2B). In particular, even the alignment of the two peaks with the main marker of EMT–namely, E-Cadherin–is preserved. This result is somewhat surprising, given that previous studies have shown that the presence of ZEB1 is crucial for EMT [29, 30]. To simulate the effect of ZEB1 knockout, we held its value fixed at -1. The landscape showed a significant shift towards the E state. This is consistent with the results of Steinway et.al [19] who showed that knocking out ZEB1, as well as other E-cadherin suppressors, decreases the probability for EMT transition.

These results indicate that while the presence of ZEB1 in the network plays a vital role in the EMT transition, its significance does not stem from the local connectivity in the double negative motif; in particular this motif is not an essential feature in giving rise to the clusters of states in the network model. We thus conclude that the bistable landscape of the ZEB1/miR-200 double negative motif in isolation is not scaled up to the network level; its effect on the steady state landscape is masked by the surrounding complexity of interactions when embedded in a large network.

To get a better sense of the importance of the ZEB/miR200 relative to other feedback loops, we subjected the network to a similar perturbation on other such motifs. No tangible change was observed in the landscape (S1a, S1b Fig in S1 File).

Experimental work has shown that ZEB2, which forms another double negative feedback loop with miR-200, also plays a vital role in EMT [8, 31]. We thus tested whether the joint action of these two double negative motifs is responsible for the two-cluster landscape. Similar to the single motif, inverting the sign of both the miR-200 to ZEB2 and the ZEB1 to miR-200 connections had no tangible effect on the landscape of states (S1b, S1c Fig in S1 File). This suggests that the details of connectivity in these local motifs is dispensable for the formation of the two clusters of states.

### Susceptibility of E/M topography to local changes in network connectivity

Although the natural suspects for local motifs supporting two clusters of states are the double negative feedback loops between miR200 and ZEB1/ZEB2, the insensitivity to manipulation of these motifs does not mean that any arbitrary local modification will not affect network properties. To address this, we varied connectivity of all local motifs in a systematic way.

**Fig 3:**
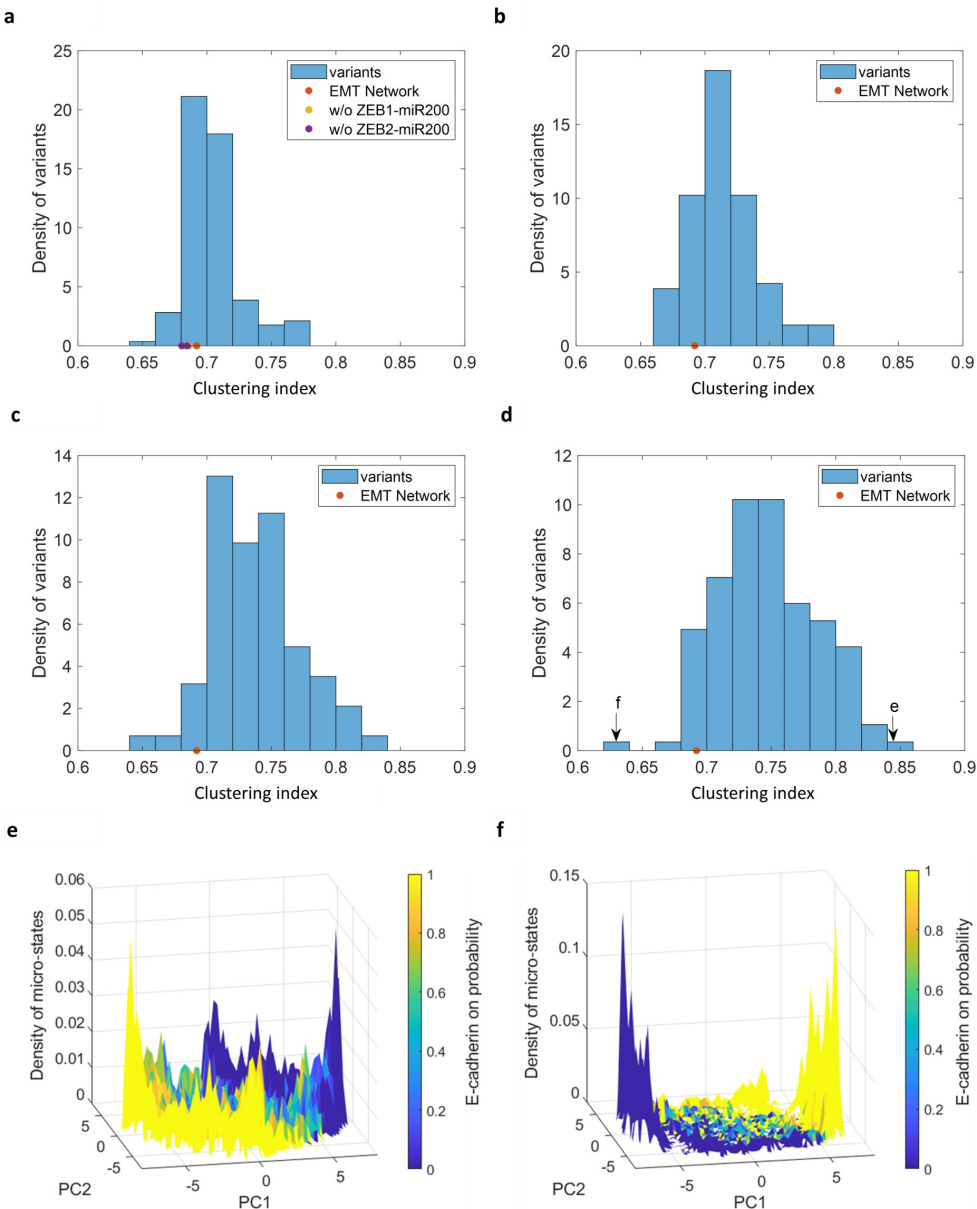
Susceptibility of the E/M topography to changes in connectivity. Following perturbations, steady states of each resultant variant network were split into two clusters using k-means, and the clustering index was computed (see main text and Methods). **(a-d)** Histograms of clustering index over ensemble of perturbations. **(a)** Inverting the sign of each of the 142 connections in the network. Dots indicate the original EMT network (orange) and the 4 variants corresponding to the inhibitory connections between miR200-ZEB1 (yellow) and miR200-ZEB2 (purple). The differences are smaller than marker size. **(b-d)** Inverting the signs of random combinations of 2 (b), 4 (c) or 6 (d) connections in the network. **(e)** Density of states corresponding to the highest clustering index (0.84, right arrow in (d)). Connections inverted: MEK-SOS/GRB2, TGFβR-TGFβ, RAF-RAS, AKT-ILK, β-Catenin(nuc)-Dest_compl and Csl-NOTCHic. Variance explained by PC1 and PC2 are 20%, 15% respectively. **(f)** Density of states corresponding to the lowest clustering index (0.63, left arrow in (d)). Connections inverted: IKK-AKT,SNAI2- β-Catenin(nuc), Dest_compl-Dest_compl, CDC42-CHD1L, NOTCHic-TrCP and ILK-SMAD. Variance explained by PC1 and PC2 are 41%, 10% respectively.

We inverted the sign of each of the 142 connections in the network, and for each perturbed network measured the degree of clustering of the resulting steady-state distribution. This was quantified using the clustering index, which is the ratio between the within-cluster variance and the total variance (see Methods for details). Low values of clustering index indicate two sharply distinct peaks. Fig 3A shows a histogram of this measure for all possible single inversions of network connections. It spans a continuous range of values, where most inversions do not change the clustering significantly. The clustering index for the original network, and the inversion of the double negative motifs described in the previous section, are depicted by colored circles in the figure (see legend). Notably, these values are located around the mean and do not have an outstanding effect relative to inversion of other connections.

Inverting the signs of more connections simultaneously (as few as 2,4 and 6 connections) results in both broadening the distribution and shifting it toward higher values of clustering index that correspond to less distinct clusters (Fig 3B–3D). Thus, changing the connectivity signs in this way tends to give rise to less clustered landscapes. Examining the landscape for specific modifications at the edges of the distribution, we find that these extreme values correspond to a flattening of the landscape (Fig 3E) or, in the other extreme, highly clustered states with very few intermediate hybrid states (Fig 3F). Thus, while not very sensitive to one or two modifications, the landscape typical of the EMT model can be destroyed by a few deliberately chosen connection modifications.

A partial explanation for the range of clustering coefficients is found by estimating the number of feedback loops with an overall positive sign [32]. These includes double negative loops, but also longer loops with positive and negative connections whose overall product is positive. We find that this number is in correlation with the clustering index, similar to previous results (S2 Fig in S1 File).

### Effects of core/periphery structure on landscape

Finding that some local changes in the signs of connectivity can distort the E/M topography while others barely affect it, motivated us to study the global structural in the network and address the questions: how do global changes in the network affect its two-cluster landscape of steady-states? Can we identify properties of the network that give rise to a large number of fixed points, and to the rugged landscape of intermediate hybrid states?

Examining the network connectivity structure on a broader scale, we see that 9 of the nodes have only outgoing edges connecting them to the rest of the network, and 8 additional ones receive input only from these nodes (both groups colored in blue in Fig 1A). Thus, the network can be decomposed into a core, where recurrent dynamics occur, and a periphery of 17 nodes that do not participate in the dynamics and remain fixed at the value of their initial condition (illustrated in Fig 5A); one can view them as external *incoming signals* through which the environment interacts with the core network. Indeed, these nodes are well identified ligands and their receptors, through which cancer cells communicate with stromal and immune cells found in their environment. They include, for instance, the epidermal growth factor (EGF), the fibroblast growth factor (FGF), the platelet-derived growth factor (PDGF) and the hepatocyte growth factor (HGF) [4, 10]. This is not a typical feature of random networks, but is likely to be present in biological systems: cellular networks are often decomposable into modules, where internal connections in a module are stronger than coupling between modules [33, 34]. It is therefore of interest to understand the effect of this aspect of network structure on dynamics and steady state landscape.

**Fig 4:**
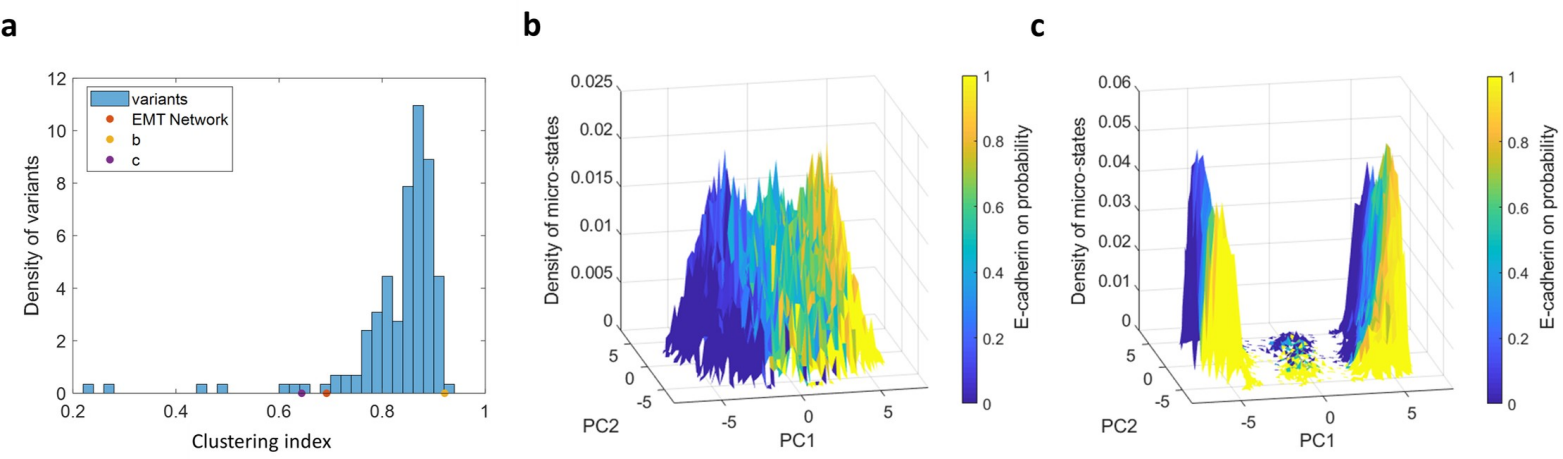
Susceptibility of E/M topography to changes in intermediate-level network structure. An ensemble of random networks was created by randomly shuffling the connections in the core network while preserving the core-periphery structure. **(a)** Distribution of clustering index. The yellow and purple dots correspond to the landscapes in (b) and (c) respectively. **(b)** Density of states near the high end of the distribution (clustering index = 0.92). Variance explained by PC1 and PC2 are 11%, 10% respectively. **(c)** Density of states near the lower tail of the distribution (clustering index = 0.64). Variance explained by PC1 and PC2 are 37%, 8% respectively.

We first simulated Boolean dynamics over an ensemble of networks with random topologies. The ensemble was created by randomly shuffling the connections of the EMT core network while preserving the connections of the inputs to the core (see METHODS for more details). The histogram of clustering index values for this ensemble spans a larger range of values than those of local changes, indicating a larger variability in the clustering topography of such networks (Fig 4A). Moreover, it is shifted toward higher values of clustering index relative to the histograms in the previous section where few local changes in connectivity were made (compare Fig 4A with Fig 3A–3D). This indicates that the specific structure of the core EMT network plays an important role in forming the two-cluster landscape. However, the effect of shuffling is highly variable: for some random topology realizations the structure is completely destroyed (Fig 4B); for a small fraction (<5%), no stable fixed points are attained; yet other core networks with a random topology can still give rise to two clusters of states in such a setting (Fig 4C). Unlike the local perturbations above, the clustering index of these networks was not correlated with the number (or fraction) of positive feedback loops (S2e, S2f Fig in S1 File). This indicates that there might be other factors that become more dominant when shuffling the connections of the network, masking the effect of the positive feedback loops.

**Fig 5:**
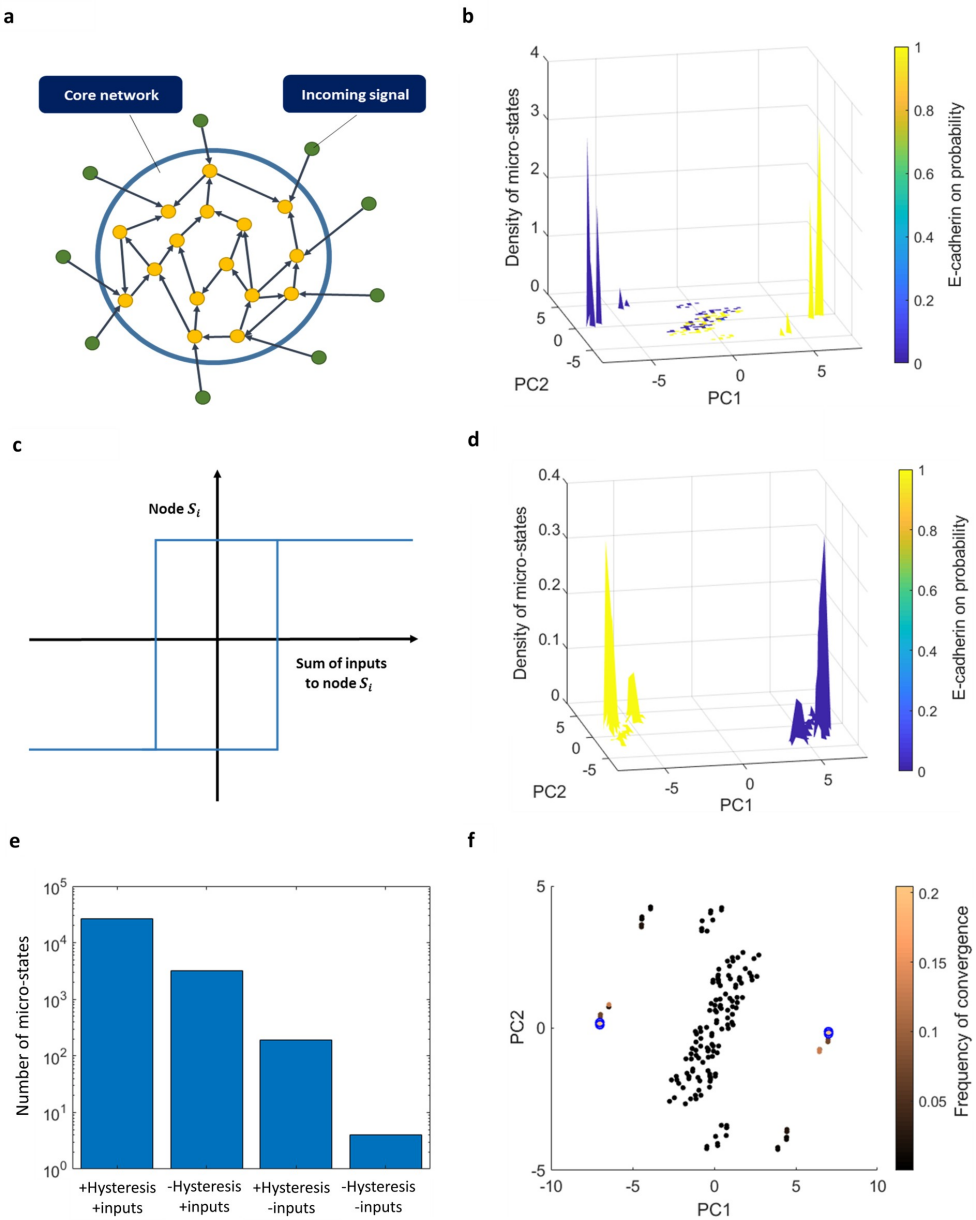
The effect of the core/periphery structure and hysteresis on the steady-state topography. **(a)** Schematic illustration of the core/periphery intermediate structure of the EMT network model. A subset of nodes (“incoming signals”) have no input, only output connections onto the “core network” (see text). **(b)** Density of the states obtained from simulating the dynamics without the inputs. **(c)** Schematic illustration of effective hysteresis in node interaction: not updating a node state in the case of zero input from its neighbors is equivalent to forming a barrier for state switching. This, in turn, leads to effective hysteresis: the threshold for switching up is different than that for switching down. This effect can be eliminated by adding noise to the connections. **(d)** Hysteresis is eliminated by adding noise to the connections: Density of the states obtained from simulating the dynamics with noise—without hysteresis. **(e)** The number of micro-states obtained by simulating the dynamics with and without hysteresis and incoming signals. The data are presented on a logarithmic scale. **(f)** PCA projection of the micro-states of the core network without the incoming signals (filled dots, see color bar for convergence frequency). Without incoming signals and without hysteresis (blue circles, *ε* = 0.001), only the high-frequency states remain.

Next, we tested the effect of the inputs by removing them from the network and simulating the dynamics on the original core network alone. Removing the inputs resulted in diluting the entire landscape (Fig 5B); Altogether removing the inputs caused around a hundredfold decrease in the number of micro-states (Fig 5E, “-Inputs”). The Biological interpretation is that multiple combinations of the incoming signals can drive the system to similar micro-states of the core network. This also implies that incorporating the state of the periphery nodes in the enumeration of the number of distinct network states results in many variations of the steady states, forming clusters of similar states. Specifically, out of the 17 input nodes 9 are independently determined by initial condition, causing each state of the core network to be spread out to 2^9^≈10^3^ states of the core+periphery. These states are affected both by the initial condition of the core and by the inputs in a noisy manner. For instance, some inputs drive the system to a certain state almost surely regardless of the initial state of the core; whereas, initial conditions located closer to the E state tend to stay in this state (Supplementary text and S3 Fig in S1 File).

### Hysteresis in gene response increases number of intermediate states

We next focused on the effect of the Boolean dynamics at the single gene level on the global network landscape. The Boolean majority rule is implemented with a "tie" (net input = 0) inducing no change; since the network is relatively sparse, the tie situation is not rare, and freezing of nodes occurs frequently. The majority rule with few inputs, therefore, effectively becomes a form of hysteresis, where a threshold needs to be crossed to modify the nodes (illustrated in Fig 5C). This leads to the fixation of many nodes for a prolonged time, which in turn increases the number of stable micro-states.

To quantify the effect of hysteresis at the single-gene level on the global network landscape, we incorporated noise to the connectivity matrix *J*. The majority rule in this case becomes

$$s_{i}(t+1)=sign\left(\sum_{j}{(J}_{ij}+{T_{ij}\cdot \xi }_{ij})\;s_{j}(t)\right)$$ (2)

where *ξ*_*ij*_ are independent Gaussian random variables with 〈*ξ*_*ij*_〉 = 0, *σ*(*ξ*_*ij*_) = *ε*, and *T* is the adjacency matrix that indicates whether a connection exists between each two nodes. This perturbation amounts to jittering the existing connections around their strict binary values of *J*_*ij*_ = ±1, but not adding or deleting connections. Even a small noise amplitude is sufficient to prevent exact ties and the fixation of nodes in a certain state. Remarkably, we found that eliminating hysteresis in this way leads to the vanishing of all the intermediate states (Fig 5D). It also leads to a tenfold decrease in the total number of steady-states obtained from simulations (Fig 5E,”-Hysteresis”). Eliminating both the effect of inputs by removing them and the effect of hysteresis by incorporating noise leaves only a handful of remaining micro-states (Fig 5E, “-Hysteresis -Inputs”). Interestingly, the more stable states, namely those with high convergence frequencies, tend to stay after incorporating noise (Fig 5F). These micro-states correspond to the fixed points of the core network that form the scaffold of the landscape topography. Due to the symmetry of dynamics with respect to flipping the sign of the states, the landscape obtained is still symmetric, so that for each state there is a corresponding state with an opposite sign.

The emergent picture from these observations is that the EMT network should be viewed as a core network that interacts with incoming signals found in the environment. The core network has only a handful of pairs of opposite-sign micro-states. This symmetry could be biologically plausible when considering binary traits such as the epithelial and mesenchymal phenotypes. The incoming signals drive the core network to spread around the two main steady-states, creating two clusters of states. The incorporation of a threshold in which the nodes retain their previous states leads to fixing the core network in intermediate states while they are on their way from the epithelial to the mesenchymal state or vice versa. This threshold can be viewed as an epigenetic barrier, such as a more restrictive chromatin state, that makes it harder for transcription factors to induce the expression of other genes [35].

In summary, our analysis of the Boolean model proposed for EMT shows that incoming signals and hysteresis are the main underlying causes for the two clusters of states observed, while details of connectivity, such as the double negative motif, are less influential at the global scale.

### Relation to experimental data

Until now we have considered general properties of the network model such as the steady-state topography, existence of two clusters and intermediate hybrid states. Although mathematically there may be other networks that give rise to these features, the EMT network model was constructed based on many detailed biological experiments. We thus expect a better match of the specific expression pattern from the original network to experimental data compared to perturbed networks. To check whether this is the case, we compared the two opposing steady-states of the network to measurements of identified cell types from the human tissues provided by the GTEx project [36]. Skin and fibroblast datasets were compared to the E and M states respectively; the data for each tissue was binarized following [17].

**Fig 6:**
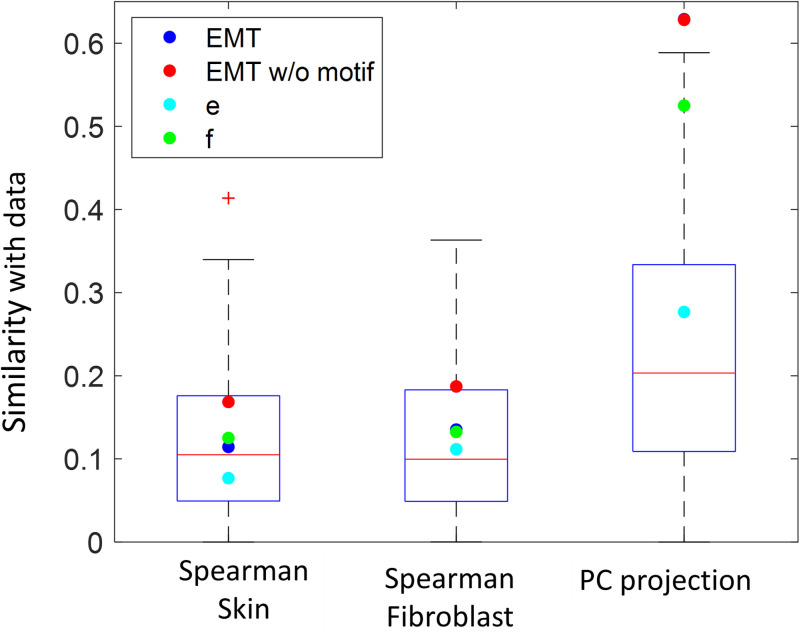
Comparison of the EMT network and perturbed networks steady states in matching to experimental data. An ensemble of random networks was created by randomly shuffling the connections in the core network while preserving the connections with inputs. EMT network and its variant with the inverted double negative motif (blue and red dots respectively) compared to the ensemble of random networks (boxplots) and the two networks in Fig 3E and 3F (these networks gave rise to the highest and lowest clustering index as a result of sign inversions) in terms of similarity to experimental data. For each network, steady states were split into two clusters using K-means. The two clusters were compared to Skin (left) and Fibroblast (middle) samples using Spearman correlation. As an alternative measure, the projection (dot product) of the first PC obtained from the steady states of each network on the first PC obtained from all the 11688 tissues available in the GTEx database (right boxplot). All measures are presented in absolute values. The differences between the blue and red dots in PC projection are smaller than marker size.

We compared the experimental data with the steady-states of the original EMT model, of the variant without the double-negative feedback loop, and of the ensemble of random networks (shuffled core connections, preserving input structure). We used two different measures–Spearman correlation and PCA-based. Fig 6 shows the Spearman correlation between models and data. Using this measure, there is no discernable difference between the modified network, random networks and the original one.

As a second measure we considered the projection of the first PC of the micro-states on that of the data. The first PC is the direction that captures the highest variance in gene expression space, and its coefficients indicate the relative contribution of the genes to that direction. Thus, models that more resemble data are expected to exhibit a larger projection. The first PC of the data was computed based on all the 11688 tissues available in GTEx database. We also computed the PC coefficients of the states arising from each of the model networks. This analysis revealed a significantly better match of the original EMT model to the data (Fig 6, right). The elimination of the double-negative motif, however, does not affect this match, once again indicating its smaller role within the global network context.

## Discussion

Understanding how network structure and dynamics give rise to biological phenotypes at a higher level of organization is a central goal of biophysics and systems biology. Many specific systems have been modeled as networks that are faithful to molecular details; at the same time random networks have been used as more abstract models. In between these two extremes, the question of what properties of a complex network give rise to its emergent behavior remains a largely open one.

In this work, we used a Boolean model of the EMT network to study the role of several local and global features on emergent network properties. This entailed defining various measures of these properties on the one hand, and various perturbations of the network on the other. Our results show that no single connection or local motif is by itself responsible for the steady state landscape of the network. Instead, we highlight several, mostly global, features of the network that underly network properties. These features are both of a statistical nature, and specific to this biological network.

The emergent properties relevant to the E/M switch include a large number of stable micro-states, organized in two clusters corresponding to the E/M phenotypes; the existence of a rugged landscape of less stable intermediates, presumably corresponding to the hybrid phenotypes; and the specific match between the model’s steady states and gene expression data.

The ZEB1/miR-200 double negative motif is often attributed as the source of the bistable structure of states in EMT [5, 6].Modifying the connection signs in this motif destroys its local functionality as a bistable switch, but has negligible effect on the global bistable landscape. In line with our computational result, experiments have demonstrated that disrupting the miR-200/ZEB1 double negative motif via CRISPR did not prevent cells from undergoing EMT [37], indicating that other interactions might also lead to this switch.

Nevertheless, previous work has established the significance of the two nodes that participate in the double-negative motif, ZEB1 and miR200. Experiments show that mesenchymal cells tend to have a high level of ZEB1 and a low level of miR200 [38, 39]. Moreover, while SNAIL can initiate E-cadherin inhibition, ZEB1 is crucial for its complete inhibition [30, 39]. Similarly, complete reversal of EMT requires suppressing EMT-inducing signal and ZEB1 [29]. The importance of ZEB1 itself is evident in our model, because although changing the connections of the ZEB1/miR200 double negative motif does not change the landscape of states (Fig 2B), a knockdown of ZEB1 resulted in a shift towards the epithelial state (Fig 2C).

Our results support the view that the significance of ZEB1 to EMT does not stem from its participation in the miR200/ZEB1 double-negative motif, but rather from its more global network properties. This view is consistent with a host of recent experimental results. ZEB1 was shown to have multiple connections to other feedback loops in the network, such as with GRHL2, OVOL2 and ESRP1 [40, 41]. The set of genes inhibited during EMT is enriched with genes that contain ZEB1 binding sites in their promoters [42]. Recent experiments [43] showed that ZEB1 is a hub in the EMT network that regulates and is regulated by multiple feedback loops between transcription factors and miRNAs. Moreover, while ZEB1 is traditionally considered a transcriptional repressor of epithelial genes, it was shown to turn into a transcriptional activator by interacting with YAP1 [44]. The ZEB1/miR-200 motif is also not uniquely dedicated to the EMT process. The results in [43] are in line with the view of this motif as also affecting other cell-state transitions [8]. These observations stress the multiplicity of roles ZEB1 can take and the intricate nature of interactions that involve it. Taken together, these experimental results together with our computational results, suggest that the cruciality of ZEB1 to EMT reflects its role as a flexible hub in the network rather than the importance of the specific mutual inhibition with miR200.

It is interesting to compare the weak influence of the ZEB1/miR-200 double negative motif to the characterization by stable network motifs [18, 19]. These are groups of nodes which, when taking on their values in the E/M state, guarantee the stabilization of these respective network states. One of the M-stabilizing motifs includes the ZEB1/miR200 double negative loop. This, however, does not contradict our result regarding its necessity. First, these motifs were defined as properties of initial conditions that lead to convergence to an E/M attractor. As such they characterize basins of attraction, rather than our use of the word motif for feedback loops in connectivity. Second, [18] defined 3 M-stabilizing motifs, implying that there are alternative ways to guarantee converging the system to the M state. Moreover, the E-stabilizing motif defined by [19] includes 66% of the nodes in network and does not include the ZEB1/miR200 motif, emphasizing that this state of the network is not defined locally.

We used the computational model to investigate many individual network perturbations and to assess their effect on the steady-state landscape. In general, the manipulation of all single connections results in a continuous and small effect on the landscape. Importantly, the effect of perturbations can go both ways: either to a more clustered or to a more smeared landscape. The original network model, constructed from experimental data [18], does not display a maximally binary landscape but rather two clusters composed of many micro-states with less stable intermediate states. Interestingly, a similar result, namely that perturbations can either increase of decrease the degree of clustering, was obtained with different network models and a different clustering criterion [32]. In further agreement with this recent work, we found that over the ensemble of perturbed networks the clustering index is correlated with the total number of positive loops in the network.

Simultaneously modifying several connections has a qualitatively similar effect but with a larger amplitude: they can ruin the two-cluster landscape, but can also sharpen the clusters, and in general span a broader range of effects. These modifications still largely maintain the statistics of connectivity while disrupting the specific local motifs derived from biology.

Examining the network on a more global scale, we found that a crucial ingredient affecting the landscape is the core/periphery organization of the network. Peripheral nodes are incoming signals that constitute redundant combinatorial pathways for inducing transitions between the stable phenotypes. While this is clearly a simplification of the model, as in reality inputs may be connected to other parts of the cell that are outside the model, it provides an approximate description of a real biological situation–the weak coupling of the EMT network to other parts of the cell. In this respect, it is important to understand the effect of such structure on the landscape.

Without the incoming peripheral nodes, a bistable landscape remains but the number of microstates drops dramatically, and two sharp peaks remain. Thus we conclude that the peripheral inputs add variation to the limited number of states of the core network. On the other hand, leaving the core-periphery structure with a randomized core creates an ensemble of networks with a broad range of landscapes, suggesting that that the core network itself has special properties. Very recent work [45] has suggested that the landscape of biological networks is related to “frustration” of states in a Boolean network (number of edges with *J*_*ij*_*s*_*i*_*s*_*j*_<0); this property might distinguish them from random networks with similar statistical properties.

Hysteresis at the single gene level also has a global effect on network dynamics. It results in fixing the genes in the same state for a prolonged time, thus forming a rugged landscape of intermediate hybrid states. These two factors–core/periphery structure and hysteresis—are necessary but not sufficient for the landscape formation, because random networks under these constraints do not necessarily give rise to two clusters of states with a smear of intermediate hybrid states.

### Limitations of the network model

A network model is always a simplification of the biological system. Specifically, drawing conclusions from a Boolean model of the EMT network entails some additional limitations. First, the Boolean dynamics is simplistic and does not allow genes to take continuous values, which might cause artifactual results. To address this limitation, we simulated continuous dynamics of the EMT network, and obtained very similar results (Supplementary text and S4 Fig in S1 File). This validates the results obtained from the Boolean dynamics.

Second, although the analyzed EMT network is based on experimental data, it is still a highly simplified model that includes only a partial set of all relevant interactions. In particular, the periphery genes do receive input from the core genes and from the environment. For instance, TGFβ can synergize with EGF ligand to induce EMT [4]. Taube et al [42] showed that while TGFβ induces the expression of EMT-inducing transcription factors, they, in turn, increase the expression of TGFβ, forming a feedback loop. Moreover, changes in the extracellular matrix, that result from tissue stress, can cause TGFβ to convert from an inactive to active form, thus promoting EMT [46]. However, it is possible that these interactions occur at slower timescales that enable decoupling of the input dynamics from the rest of the network. Despite these limitations, the EMT network model still captures experimental data better than other typical random network, justifying its use (Fig 6).

Third, the Boolean nature of the connectivity matrix *J* may not be suitable to describe variations in coupling strengths between the genes. The contribution of the mutual inhibition of ZEB1 and miR200 to the formation of two clusters of states may be due to particularly strong couplings between these components or between them and the rest of the system. Under the Boolean setting, this effect is washed away. Note, however, that this model does capture the effect of ZEB1 knockdown, possibly reducing the concern regarding this aspect.

In summary, our results highlight the notion that phenotypic traits may not be the result of interactions between a small set of genes but rather of complex integration of regulatory interactions spread over the network. This is in line with the view that complex traits entail association signals spread all over the genome, including genes that are not considered directly related to the trait [47]. Nevertheless, the modular structure of the entire cellular network, which manifests itself for the EMT network as a core/periphery structure, has an important role in shaping the landscape of two clustered states with hybrid intermediates. It remains an open question how the participating genes and the entire network shape the landscape, and support the control of multiple switches simultaneously, while coordinating between them.

## Methods

### Simulations

All simulations were performed using scripts written in Matlab (MathWorks, MA, USA). The landscape of states was constructed by performing 38,000 simulations of the Boolean model (unless explicitly stated otherwise). Each simulation started from a random initial condition of the network state and the nodes were updated asynchronously for 1500 time steps. Network states that did not change over the last 500 time steps were considered stable and were projected on a two-dimensional map using principal component analysis (PCA). The map was binned to 4624 bins, and the states in each bin were colored by the fraction of steady states that express E-cadherin within the same bin.

#### Core/Periphery decomposition

To decompose the network into a core and a periphery, we pruned the network by removing the nodes that receive no input and all the downstream nodes that receive input only from these nodes. The 17 removed nodes composed the periphery and the remaining 55 nodes constituted the core network in which all the nodes receive an input. The core network contains 35 inhibiting connections and 76 promoting connections.

#### Construction of a random network ensemble

Random networks were constructed by randomly redistributing the connections of the core while preserving the number of inhibiting and promoting connections. The connections of the peripheral nodes with the core and with themselves were not changed. Thus, each random network contained a random core that have same number of promoting and inhibiting connections as the EMT network, and a periphery that is the same as the EMT network. Less than 5% of the simulated random networks did not give rise to stable steady states.

#### Clustering index

The steady states of each network were split into two clusters using k-means algorithm. The clustering of the steady states was quantified by the ratio between the sum of within-cluster distances and the sum of all distances from the steady states’ mean:

$$Clustering\;index=\frac{\sum_{i\in C_{1}}{\left\|S_{i}-\mu_{1}\right\|}_{2}^{2}+\sum_{i\in C_{2}}{\left\|S_{i}-\mu_{2}\right\|}_{2}^{2}}{\sum_{i}{\left\|S_{i}-\mu_{tot}\right\|}_{2}^{2}}$$ (3)

where μ_1_ and μ_2_ are the centroids of the clusters *C*_1_ and *C*_2_ respectively. μ_*tot*_ is the mean of all steady states. *S*_*i*_ is a steady state. The distances were computed in the full 72-dimensional gene expression space. In general we found that our conclusions were not sensitive to the score chosen, and other measures behaved similarly.

#### Calculating the number of positive feedback loops

The number of positive feedback loops was estimated as in [32] using the network module in Python 3.7. A feedback loop is defined as the directed path traversed along the edges of the network whose first and last nodes are the same. The sign of a loop is determined by the product of the signs of the edges that constitute the loop. For instance, ZEB-miR200-ZEB is a positive feedback loop, as it goes through 2 inhibitory edges.

#### Experimental data analysis

Data in Fig 6 belong to the GTEx project [36] and were downloaded from the GTEx portal (https://gtexportal.org/home/datasets). This database includes RNA-seq measurements of 11688 tissues. We followed the procedure of [17] to binarize the data. Briefly, for each gene the distribution of expression values in samples labeled as “Cells–Transformed Fibroblasts” and “Skin–Not Sun Exposed (Suprapubic)” was examined. A threshold was set for each gene to maximally differentiate these classes, and this defined a binary expression level for all genes and all tissues.

To test how well the EMT network captures experimental data, we compared the similarity of the experimental data to the steady states obtained from the EMT network, the EMT network w/o motif and 142 random networks that preserve the structure of core/periphery using two measures.

1. Spearman correlation between the mean gene expression of the “Skin–Not Sun Exposed (Suprapubic)” tissues and the mean gene expression of one of the clusters of the steady states that were clustered using K-means.
2. The projection of the first PC of the steady states of each network over the first PC obtained from the data (PCA was performed on all tissues. Similar results were obtained when using only skin and fibroblasts).

## Supporting information

S1 File (DOCX)
