# Supplementary material for: Local and global features of genetic networks supporting a phenotypic switch
Source: PLoS One. 2020 Sep 3;15(9):e0238433. doi: 10.1371/journal.pone.0238433 (PMC7470255; doi:10.1371/journal.pone.0238433)
Supplement: S1 File — (DOCX) [file pone.0238433.s001.docx]

**Supplementary Information for**

**Local and global features of genetic networks supporting a phenotypic switch**

**Aseel Shomar, Omri Barak, Naama Brenner**

This PDF file includes:

Supplementary text

Figs. S1-S4


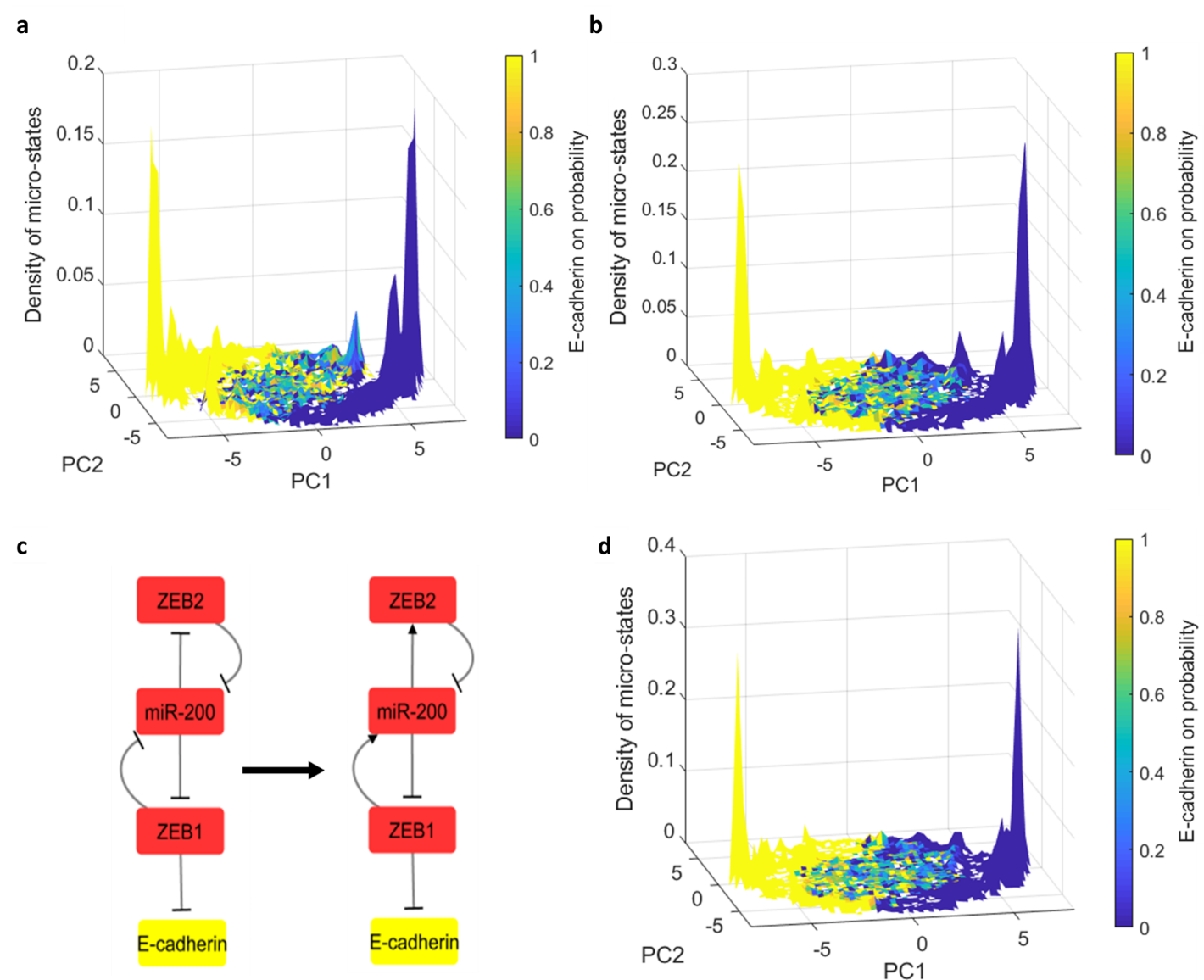


**Fig. S1. The effect of double negative motifs on the emergence of two clusters of states.** (a) The density of microstates obtained after eliminating the catenin-nuc/catenin-mem motif. This is a double negative feedback loop between catenin-mem and catenin-nuc (where the nodes are two different localization states - membrane and nucleus). We inverted the sign of the connection from catenin-nuc to catenin-mem. (b) The density of microstates obtained after eliminating the ZEB2/miR200 motif by turning the inhibitory connection from miR-200 to ZEB2 into a promoting connection. (c) Left: the two coupled double negative motifs ZEB1/ miR-200 and ZEB2/miR-200. ZEB1 inhibits E-cadherin. Right: the inhibitory connection from ZEB1 to miR-200 was turned into a promoting connection, and the inhibitory connection from miR-200 to ZEB2 was turned into a promoting connection. This eliminates both double negative motifs and breaks the symmetry between them. (d) The density of microstates obtained after eliminating the two double negative motifs as in (c).


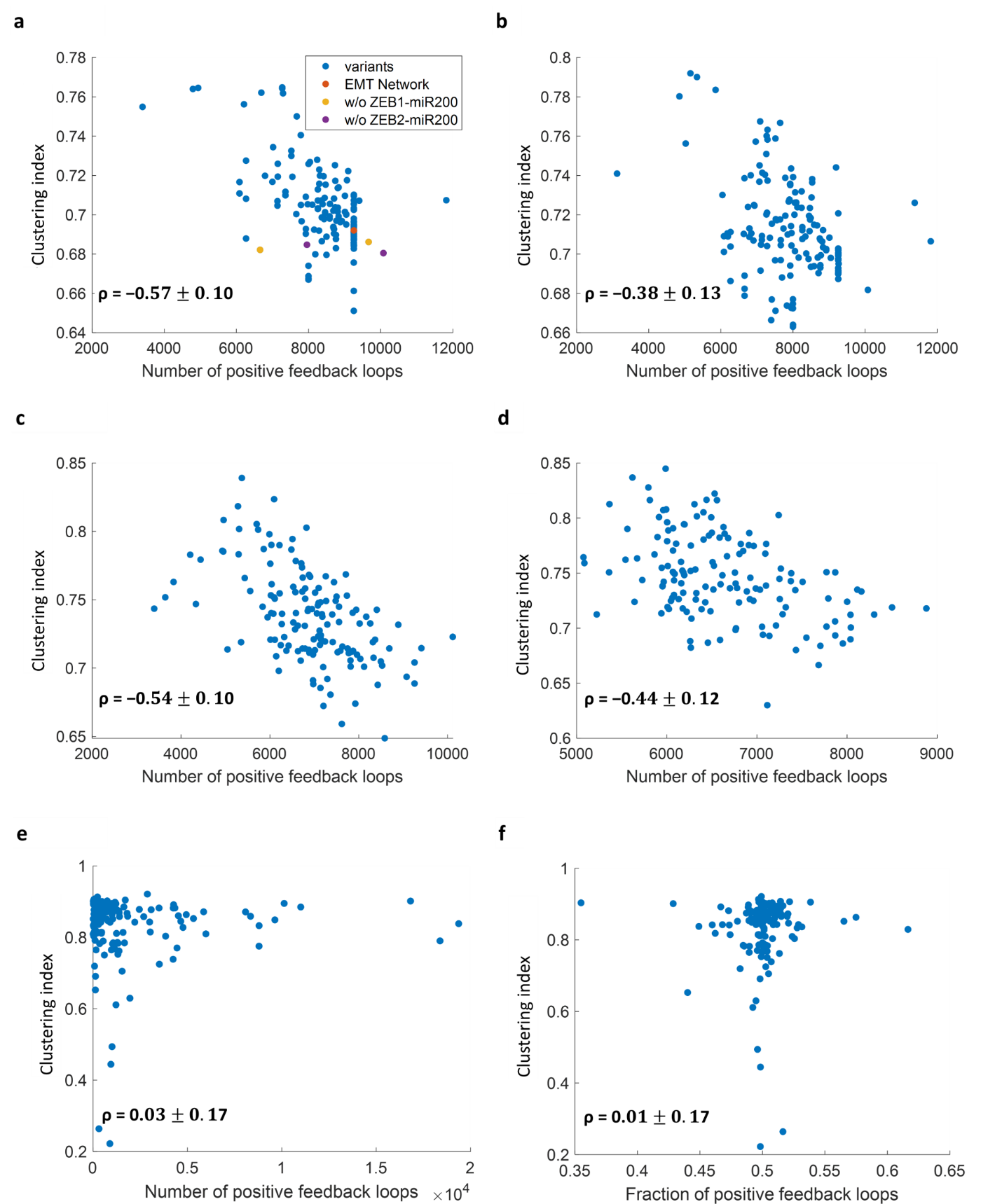


**Figure S2. Effect of positive feedback loops on the emergence of two clusters of states.** **(a-d)** Clustering index vs. the number of positive feedback loops for (a) single inversions, (b) two inversions, (c) four inversions and (d) six inversions. **(e)** Same as **(a-d)** for random networks that preserve the core/periphery structure. **(f)** Clustering index vs. fraction of positive feedback loop out of all loops for random networks. $\rho$ is Pearson’s correlation.

**Supplementary text**

***The effect of incoming signals and initial condition on final steady-state***

Can incoming signals drive the transition of the core network from the epithelial to mesenchymal phenotype and vice versa?

To answer this question, we simulated the dynamics for many initial conditions of the core network ($x_{j}\in\left\{ \pm1 \right\}^{55}$) over all possible combinations of incoming signals ($y_{i}\in\left\{ \pm1 \right\}^{9}$). Each such simulation converged to a steady state of the core network $z_{ij}\in\left\{ \pm1 \right\}^{55}$, allowing us to define an average state associated with the *i*-th input,  $\overline{z_{i}}=\langle z_{ij}\rangle_{j}$. We characterized the location of any network state $x$ along the E-M axis by its distances to the 'centers of mass' of each phenotype cluster: $d\left( x \right)=\frac{d\left( x,E \right)}{d\left( x,E \right)+d(x,M)}$.

We find that incoming signals can drive the transition of the core network from one phenotype to the other, but this transition is dependent on the initial condition. The final (steady state) locations along the axis are presented in Figure S3a (indexed by color) for various initial conditions (X axis) and incoming signals (Y axis). The figure shows that both the initial conditions and the incoming signals affect the final state. For instance, some incoming signals drive the system to a certain state almost regardless of the position of the initial state along the E-M axis; whereas, initial conditions located closer to the E state of the axis tend to stay in this state. This is also manifested by the higher fraction of E-cadherin expressing states obtained for more epithelial initial conditions (Fig.S3b).


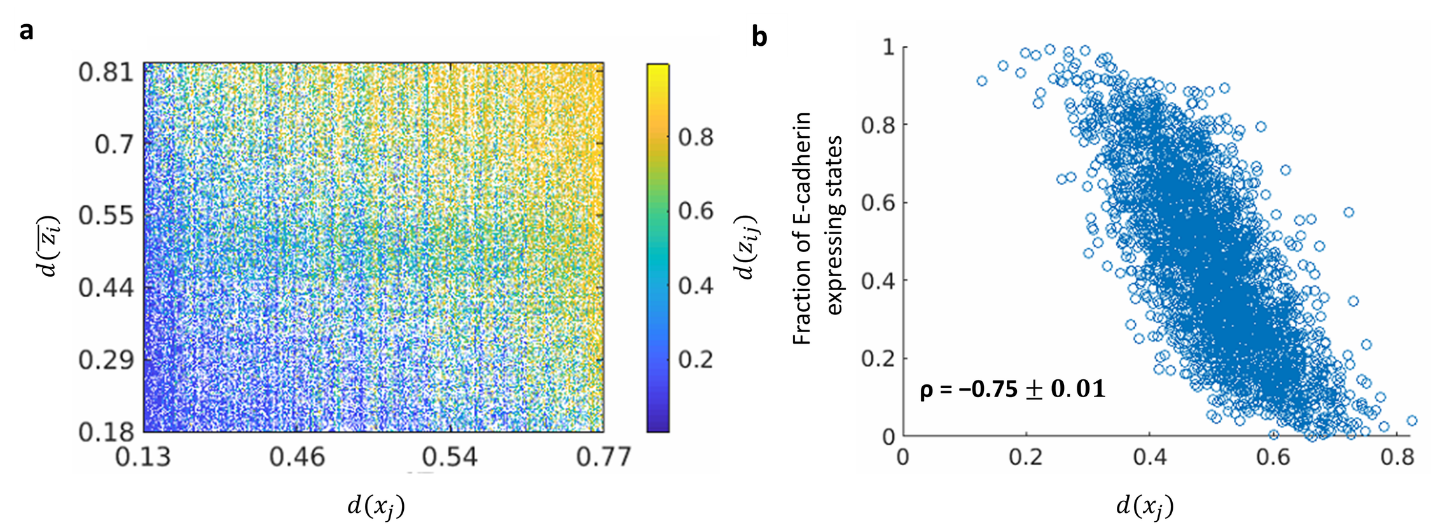


**Fig S3. Incoming signals and initial conditions drive the core network from one phenotype to the other.** (a) The final steady state, $d(Z_{ij})$, as a function of initial condition (x-axis) and incoming signals (y-axis). All states are characterized by their location on the E-M axis (see text). Each combination of incoming signals is presented by the average location of the steady states along the axis over initial conditions. (b) The fraction of E-cadherin expressing states and the E-M location of the initial condition are inversely correlated. $\rho$is Pearson’s correlation.

**Continuous dynamical model of the EMT network**

Despite the usefulness of Boolean models, confining gene expression to only two values $\left\{ \pm1 \right\}$ is simplistic. To test the sensitivity of our results to this simplification, we simulated a continuous model of the EMT network. The continuous update rule is:

$\left\{ \begin{aligned} s_{i}\left( t+1 \right)=\tanh\left( \frac{\sum_{j} J_{ij}s_{j}\left( t \right)}{\mu} \right) if \left| \sum_{j} J_{ij}s_{j}\left( t \right) \right|>b \\ s_{i}\left( t+1 \right)=s_{i}\left( t \right) if \left| \sum_{j} J_{ij}s_{j}\left( t \right) \right|\leq b \end{aligned} \right.$ (1)

where $b$ is the threshold of hysteresis and $\mu$ is the sigmoid steepness, smaller values being steeper (Fig. S4a). Simulating these dynamics over 39,000 initial conditions, until the network reaches a steady state, yields two clusters of micro-states corresponding to E and M states and an intermediate valley of hybrid states (compare Fig. S4b with Fig. 1c). Turning the inhibitory connection from ZEB1 to miR-200 into a promoting connection, resulted in no change in the landscape of states, while a knockdown of ZEB1 led to a significant bias towards the E state (compare Fig. S4c-d with Fig. Fig. 2b-c). These results are congruent with those obtained for the Boolean model, indicating that the discreteness of the Boolean model does not affect the results.


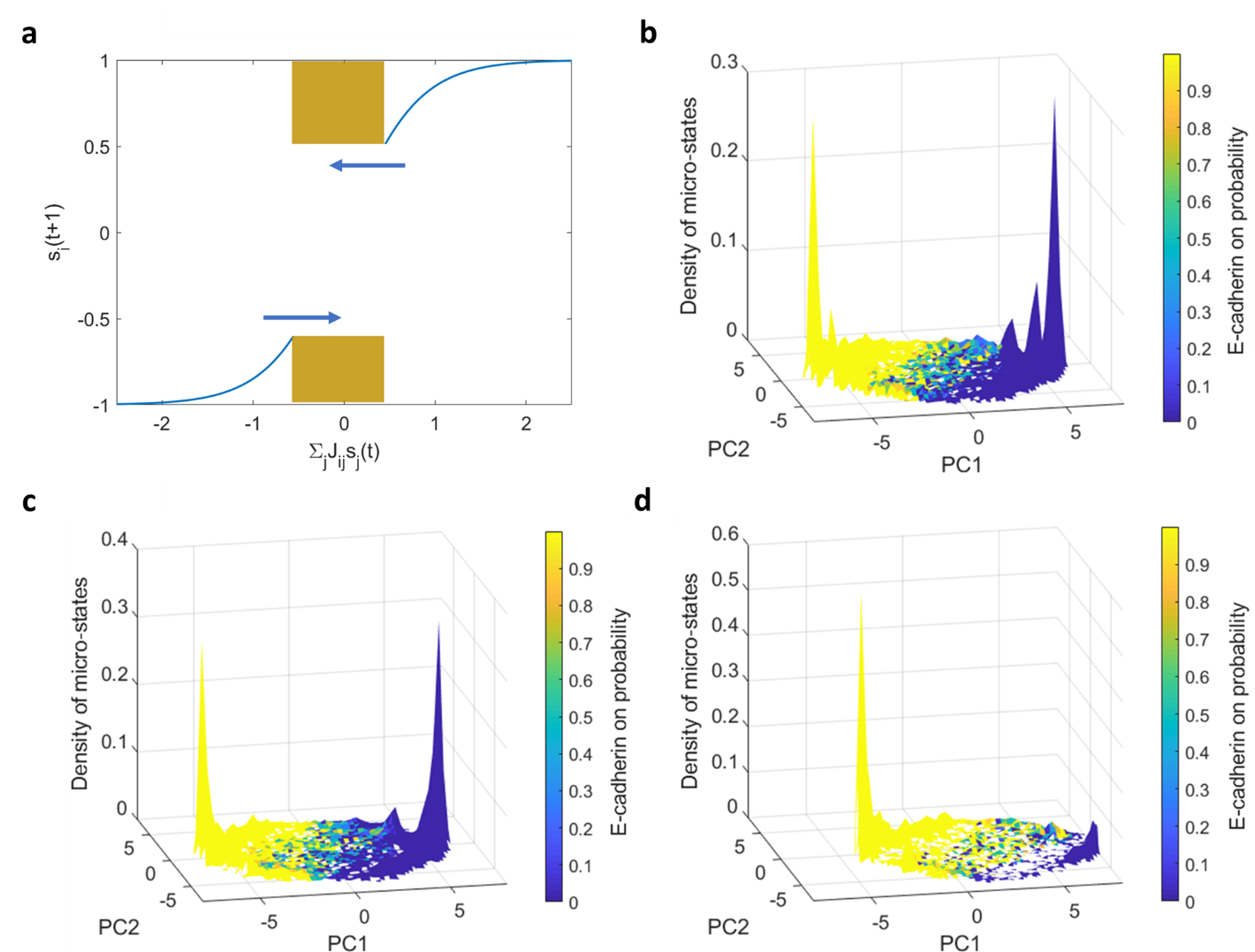


**Fig. S4. The density of states for continuous dynamics.** (a) The continuous update rule for $b=0.5$ and $\mu=0.8$. If the sum of interactions, $\sum_{j} J_{ij}s_{j}\left( t \right)$, falls within the dark yellow region, the node $i$ preserves its previous state. If the sum of interactions, $\sum_{j} J_{ij}s_{j}\left( t \right)$, falls outside the dark yellow region, the node $i$ is updated according to the blue sigmoidal curve. (b) The density of micro-states obtained from the continuous model for the EMT network. (c) The density of micro-states obtained from the continuous model after eliminating the double negative motif by turning the inhibitory connection from ZEB1 to miR-200 in the EMT network into a promoting connection. No tangible changes are observed. (d) Following ZEB1 knockdown, the landscape obtained from the continuous model exhibits a strong shift towards the E states.
